# Chemically Engineering Magnetic Anisotropy of 2D Metalloporphyrin

**DOI:** 10.1002/advs.201700019

**Published:** 2017-07-18

**Authors:** Peng Wang, Xue Jiang, Jun Hu, Jijun Zhao

**Affiliations:** ^1^ Key Laboratory of Materials Modification by Laser Ion and Electron Beams (Dalian University of Technology) Ministry of Education Dalian 116024 China; ^2^ College of Physics Optoelectronics and Energy Soochow University Suzhou Jiangsu 215006 China

**Keywords:** 2D organic materials, chemical engineering, magnetic anisotropy, spin‐orbit coupling

## Abstract

Continuous miniaturization of magnetic units in spintronics devices inspires efforts to search for novel 2D magnetic materials with giant magnetic anisotropy energy (MAE). Through systematic first‐principles calculations, large MAE of 24 meV in W or Re embedded 2D polyporphyrin frameworks is found. Interestingly, the MAE can be enhanced up to 60 meV, through replacing the hydrogen atoms on the edges of the Re based 2D polyporphyrin framework by hydroxyl and amino radicals. Analysis of the electronic structures reveals that the enhancement of MAE is mainly attributed to charge redistributions and energy shifts of Re 5d orbitals induced by the functional radicals. The findings pave a new and feasible way for tailoring the magnetic properties of magnetic organic materials to fulfill the criteria for applications in spintronics devices at high temperature.

## 1 Introduction

Rapid development of spintronics drives endeavors to explore magnetic properties of magnetic materials with their sizes down to nanometer scale. In this realm, the magnetic anisotropy energy (MAE), which describes the energy cost to flip the spin between bistable states, is the most critical factor, because it determines the blocking temperature below which the magnetic anisotropy can prevent spin fluctuation. Unfortunately, “intrinsic” MAEs of most magnetic nanomaterials are very small, around the order of ≈1 meV,^1^ which is too small to overcome the thermal excitation energy at room temperature (≈26 meV). The MAE of hcp cobalt, which is widely used in modern hard disk, is merely 0.01 meV per atom.^2^ By alloying with other metals, the MAE can be enhanced up to 1–2 meV per atom,^3^ still far less than the required value.

It is known that magnetic anisotropy originates from the spin‐orbit coupling (SOC). Due to the highly localized d orbitals, transition metal (TM) atoms exhibit remarkable SOC effect, endowing them with great potential for achieving large magnetic anisotropy in magnetic nanomaterials containing TM atoms. In addition, ligand field plays an important role in tailoring the magnetic anisotropy.^4^ With proper substrate which provides specific ligand field, giant MAEs up to 60 meV can be achieved in TM adatoms deposited on MgO(100) surface^5^ and 2D materials such as pristine or defective graphene,^6^, ^7^, ^8^, ^9^, ^10^, ^11^, ^12^ g‐C_3_N_4_,^13^ graphyne,^14^ and MoS_2_ monolayers.^15^

Recently, 2D covalent organic frameworks (COFs)^16^ have attracted wide attention because TM atoms can be embedded into COFs, forming periodic 2D magnetic structures.^17^, ^18^ The magnetic properties especially the magnetic anisotropy of TM atom decorated 2D COFs have been investigated theoretically^17^, ^18^, ^19^, ^20^, ^21^ and experimentally.^17^, ^19^, ^20^, ^21^, ^22^ These studies imply that TM atom decorated 2D COFs are of great potential for future applications in spintronics devices, because they have several prominent advantages: (i) 2D COFs are easy to prepare with inexpensive ingredients; (ii) their geometrical configurations are basically controllable; (iii) TM atoms can be naturally and uniformly embedded into the 2D network without clustering; (iv) the magnetic coupling between the neighboring TM atoms is tunable. However, achieving giant MAEs in 2D COFs are still challenging.

To obtain giant MAEs in 2D COFs, heavy 5d TM atoms are good candidates because the SOC effects are strong. In addition, the magnetic anisotropy may be further modified through “extrinsic” approaches such as charge injection^23^ and external electric fields.^24^ However, it is delicate to control the amount of injected charge and the strength of external electric field. Therefore, we investigated the magnetic anisotropy of 5d TM decorated polyporphyrin frameworks (TM@Pp) and proposed a new feasible and reliable chemical method for the first time by modifying the functional radicals, based on systematic first‐principles calculations. We found that W@Pp and Re@Pp possess large MAE of about 24 meV. Interestingly, replacing the hydrogen atoms on the edges of the 2D Re@Pp by hydroxyl and amino radicals enhances the MAE up to 60 meV. Furthermore, Re@Pp shows robust ferromagnetic coupling. These properties imply promising applications of 2D Re@Pp in spintronics devices above room temperature. From the analysis of electronic structures, we revealed that the enhancement of MAE can be attributed to the charge redistribution due to the different electron donating capabilities of the functional radicals. Our results suggest a new means to engineer the magnetic anisotropy of 2D organic materials by chemical manipulations.

## 2 Results and Discussion

**Figure 1:**
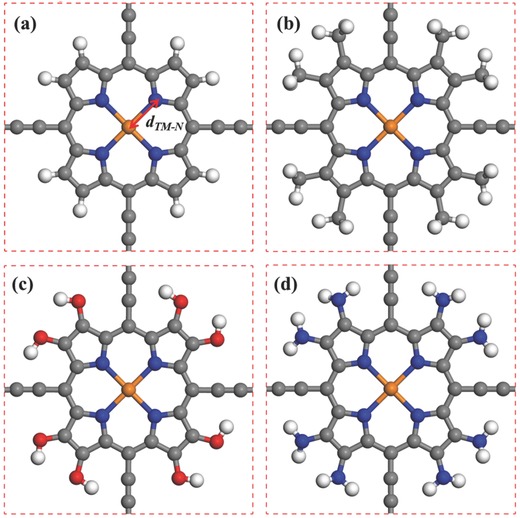
Top view of the 2D a) TM@Pp, b) TM@M‐Pp, c) TM@H‐Pp, and d) TM@A‐Pp. The gray, blue, white, red, and orange spheres stand for C, N, H, O, and TM atoms, respectively. *d*_TM‐N_ denotes the bond lengths between TM and N atoms.

The atomic structure of 5d TM@Pp is shown in **Figure** **1**a. We considered a series of 5d TM atoms (Ta, W, Re, Os, Ir) as the central atom which provides magnetic moment and magnetic anisotropy of a TM@Pp. **Table** **1** lists some key properties of TM@Pp. The bond lengths between TM and N atoms lie in the range of 2.07 ± 0.06 Å, showing strong constraint from the polyporphyrin framework. For an embedded TM atom, its binding with the polyporphyrin framework can be characterized by the adsorption energy: *E*_ad_ = *E*(TM) + *E*(Sub) − *E*(Tot). Here *E*(Tot), *E*(TM), and *E*(Sub) are the energies of TM@Pp, the individual TM atom and the pure polyporphyrin framework, respectively. For the investigated systems, the adsorption energies are in the range of 9.8–12.4 eV, manifesting strong binding between the TM atom and polyporphyrin framework (see Tables S1 and S2 of Supporting Information). This is reasonable since four strong metal‐nitrogen bonds form through the binding between the TM atom and polyporphyrin framework. In fact, large adsorption energies were also found in similar system—metal mesotetraphenylporphines where the adsorption energies vary from 6.3 to 10.8 eV for the late 3d transition metals (from Fe to Zn).^25^ For the cases in our work, the 5d transition metals are even more active than their 3d counterparts, which results in even larger adsorption energies.

**Table 1:** Structural and magnetic properties of 2D TM@Pp. Here, *d* is the distance between the transition metal and nitrogen atom. *M*_s_ and *M*_s,TM_ are the total spin moment per unit cell and the local spin moment on TM atom, respectively. MAE is the magnetic anisotropy energy per unit cell

TM	Ta	W	Re	Os	Ir
*d* [Å]	2.08	2.08	2.06	2.03	2.03
*M*_s_ [*µ*_B_]	1.9	4.0	3.0	2.0	1.0
*M*_s,TM_ [*µ*_B_]	1.3	2.7	2.8	1.3	0.6
MAE [meV]	−4.9	24	23.9	−45.9	−8.3

It can be seen from Table 1 that all considered systems are spin polarized and the spin moments are mainly localized on the central TM atoms. W and Re atoms possess largest local spin moments of 2.7 and 2.8 µ_B_, respectively, while smallest local spin moment (0.6 µ_B_) is on Ir atom. Although the SOC effect in all TM atoms is strong, only W, Re, and Os atoms induce large magnetic anisotropies when they are placed at the center of polyporphyrin framework, with MAEs of about 24 meV for W@Pp and Re@Pp and −46 meV for Os@Pp. Based on the definition (see Methods section of the Supporting Information), a negative MAE corresponds the in‐plane preferential spin orientation. In addition, the in‐plane magnetic anisotropy is very small for Os@Pp (less than 1 meV), which implies that the spin orientation of Os@Pp is undetermined even at very low temperature. On the contrary, positive MAE indicates the perpendicular preferential spin orientation without additional uncertainty for spin orientation. Therefore, large perpendicular magnetic anisotropy—corresponding to large positive MAE—is beneficial for applications in spintronics devices.^26^ Clearly, the MAEs of W@Pp and Re@Pp (≈24 meV) are quite large compared to most 3d magnetic nanostructures^1^ and close to the thermal excitation energy at room temperature (≈26 meV). However, the MAE is still not large enough for applications at room temperature.

The magnetic anisotropy of a magnetic nanostructure can be affected by subtle change of the ligand field around the central TM atom, due to the modification of electron occupancy around the chemical potential.^9^, ^27^ On the other hand, the highest occupied molecular orbital and lowest unoccupied molecular orbital of organic materials can be manipulated conveniently by adjusting functional radicals with different electron donating capabilities. Therefore, we replaced the H atoms on the edges of the polyporphyrin framework by methyl (—CH_3_), hydroxyl (—OH), and amino (—NH_2_) radicals, respectively, corresponding to TM@M‐Pp, TM@H‐Pp, and TM@A‐Pp, as shown in Figure 1b–d. After structural relaxation, the TM–N bond lengths change little as listed in **Table** **2**. The electron donating capability of the considered functional radicals is in the order of: hydrogen (—H) < methyl (—CH_3_) < hydroxyl (—OH) < amino (—NH_2_).^28^ Consequently, the magnetic properties of the modified TM@Pp are expected to vary with electron donating capability of the corresponding functional radicals.

**Table 2:** Geometric and magnetic properties of the modified 2D TM@Pp. Here, *d* is the distance between the metal and nitrogen atom. *M*_s_ and *M*_s,TM_ are the total spin moment per unit cell and the local spin moment on TM atom, respectively. TM@M‐Pp, TM@H‐Pp, and TM@A‐Pp refer to modified TM@Pp with the edge hydrogen atoms replaced by methyl, hydroxyl, and amino radicals, respectively

TM		Ta	W	Re	Os	Ir
TM@M‐Pp	*d* [Å]	2.10	2.09	2.08	2.06	2.05
	*M*_s_ [*µ*_B_]	1.8	4.0	3.0	2.0	1.0
	*M*_s,TM_ [*µ*_B_]	1.4	2.7	2.8	1.6	0.6
TM@H‐Pp	*d* [Å]	2.09	2.08	2.07	2.03	2.03
	*M*_s_ [*µ*_B_]	1.7	3.7	3.0	2.0	1.0
	*M*_s,TM_ [*µ*_B_]	1.3	2.5	2.5	1.6	0.6
TM@A‐Pp	*d* [Å]	2.10	2.08	2.08	2.04	2.04
	*M*_s_ [*µ*_B_]	1.5	2.9	3.0	2.0	1.0
	*M*_s,TM_ [*µ*_B_]	1.3	2.3	2.5	1.7	0.6

**Figure 2:**
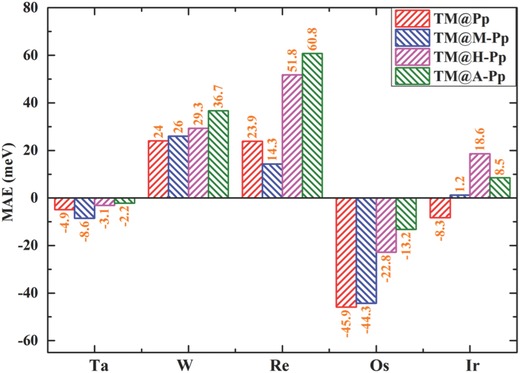
The MAEs of the original TM@Pp and the modified ones (TM@M‐Pp, TM@H‐Pp, and TM@A‐Pp).

In most cases, the total spin moments of modified TM@Pp remain nearly unchanged, but those of W@Pp change significantly because the electron occupancy increases more in minority spin states than in majority spin states (Figure S1 of Supporting Information). In addition, the local spin moment on W and Re atoms decreases notably after hydroxyl and amino modifications. *M*_s,W_ decreases to 2.5 and 2.3 µ_B_ from 2.7 µ_B_, respectively; while *M*_s,Re_ decreases to 2.5 µ_B_ from 2.8 µ_B_. Interestingly, the MAE is altered substantially for all cases, as shown in **Figure** **2**. For W@Pp and its derivatives, the MAE increases monotonously with the increasing electron donating capability of the functional radicals and reaches 36.7 meV in W@A‐Pp, with largest increment by about 50% compared to that in the original W@Pp (24 meV). More strikingly, incorporation of hydroxyl and amino radicals in Re@Pp can enhance the MAEs from 24 to 52 and 61 meV, respectively. Such giant MAEs endow the possible applications of these materials at relatively high temperature.

**Figure 3:**
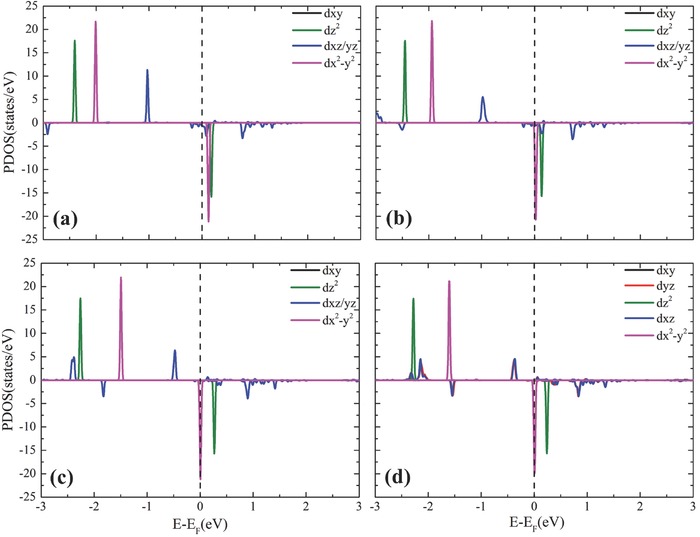
Projected density of states (PDOS) of 5d orbitals of the Re atom in a) Re@Pp, b) Re@M‐Pp, c) Re@H‐Pp, and d) Re@A‐Pp.

To gain insight into the effect of electron donating groups (EDGs) on the MAE, we plotted the projected density of states (PDOS) of the 5d orbitals of Re atoms in Re@Pp based systems in **Figure** **3**. The states of most 5d components locate near the Fermi level, while the states of *d_xy_* orbital locate about 4–5 eV higher than the Fermi level (outside the energy range in Figure 3) due to the repulsion from N 2p orbitals. It can be seen that the majority spin states of $d_{z^{2}}$, $d_{x^{2}-y^{2}}$, *d*_*xz*/*yz*_ are all occupied, while the corresponding minority spin states are unoccupied, resulting in large local magnetic moment on the Re atom. Owing to the geometrical symmetry of the polyporphrin framework, *d_xz_* and *d_yz_* states are almost twofold degenerated and are much more delocalized. After modification with hydroxyl and amino, the electron occupation on *d*_*xz*/*yz*_ decreases from 0.80 to 0.78, and 0.77, manifested by the reduction of the PDOS of *d*_*xz*/*yz*_ as shown in Figure 3. The energy levels of $d_{x^{2}-\;y^{2}}$ orbital move toward the Fermi level, leading to reduction of the exchange splitting of the majority and minority spin states. Most strikingly, the minority spin state of the $d_{x^{2}-\;y^{2}}$ orbital becomes partially occupied after modification, with electron occupations of about 0.08, 0.23, and 0.19 for methyl, hydroxyl, and amino radicals, respectively. Therefore, the local spin moment on Re atom reduces compared to the unmodified system (see Table 2). Obviously, modifying functional radicals is an effective way to engineer the electron occupancy and hence tailor the MAE.

To further elucidate the mechanism of tuning MAE, we calculated the MAEs of the original and modified Re@Pp using the Torque method,^27^, ^29^ where the MAE can be expressed as

(1) $$\mathrm{MAE}\;=\;\;\sum_{i\in \mathrm{occ}}{\left\langle \Psi_{i}|\frac{\partial H_{\mathrm{SO}}}{\partial \theta }|\Psi_{i}\right\rangle }_{\theta \;=\;45^{{}^{\circ}}}$$

**Figure 4:**
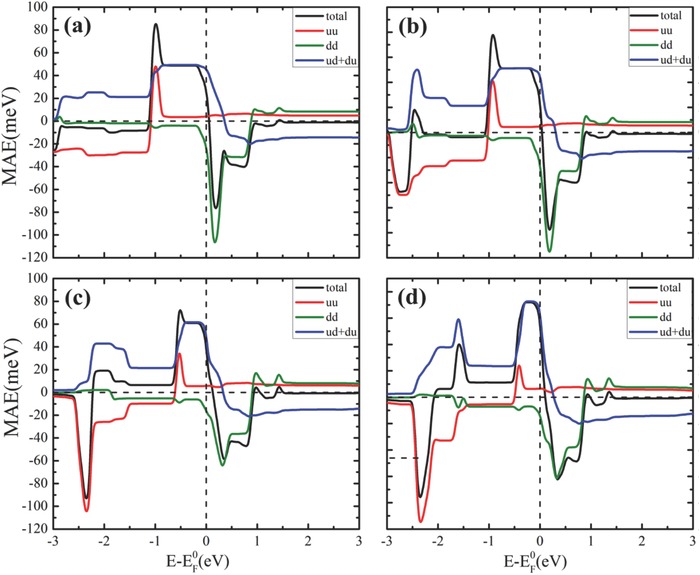
Fermi‐level dependence of the total and decomposed MAEs of a) Re@Pp, b) Re@M‐Pp, c) Re@H‐Pp, and d) Re@A‐Pp. Here, “uu,” “dd,” and “ud+du” notate the contributions from the coupling between majority spin channels, between minority spin channels, and between crossover spin channels, respectively. $E_{F}^{0}\;$ stands for the natural Fermi level.

Here θ is the polar angle of spin angular momentum relative to the normal of the polyporphyrin framework, *Ψ_i_* is the *i*th relativistic eigenvector, and *H*_SO_ is the SOC Hamiltonian. Within the rigid band model, the Fermi level is allowed to deviate from the natural Fermi level. As seen from **Figure** **4**, for both the original and modified Re@Pp, the coupling between crossover spin channels (ud+du term) dominates near the Fermi level and is the main origin of the positive MAEs. In contrast, the coupling between minority spin channels (dd term) leads to huge negative contribution to the MAEs above the Fermi level, resulting in sharp drop of the MAE above the Fermi level. This feature implies that the MAE can be turned easily from large positive into large negative by injecting extraelectron, which offers extra degree of freedom to engineer the MAE of these systems.

From Figure 4, it can be seen that the plateau right below the Fermi level of the MAE (ud+du curves) becomes narrower in width and higher in height as the electron donating capacity of the functional radicals increases, which leads to significant enhancement of the total MAE, especially for hydroxyl and amino. To reveal the origin of this effect, we decomposed the contribution from each pair of states involved in the SOC Hamiltonian, based on the second perturbation theory where the MAE(ud+du) is expressed as^30^

(2) $$E_{x}-E_{z}\;\approx \;-\xi^{2}\;\sum_{u,\;o}\left[\frac{{\left|\left\langle o|L_{z}|u\right\rangle \right|}^{2}}{E_{u}-E_{o}}\;-\;\frac{{\left|\left\langle o|L_{x}|u\right\rangle \right|}^{2}}{E_{u}-E_{o}}\right]$$

Here ξ stands for the SOC constant; *E*_o_ and *E*_u_ denote the energy levels of the occupied and unoccupied states, respectively; *L_z_* and *L_x_* are the *z* and *x* components of angular momentum operator. We found that the contribution from *L_z_* [i.e., the first term in Equation (2)] is negligible, while that from *L_x_* dominates. The coupling between *d*_*xz*/*yz*_ in majority spin channel and $d_{x^{2}-y^{2}}$ and $d_{z^{2}}$ in minority spin channel through *L_x_* [i.e., the sencond term in Equation (2)] contributes to large positive MAE(ud+du) and hence to the total MAE. Furthermore, the distance between the corresponding energy levels, i.e., *E*_u_ − *E*_o_ in Equation (2), reduces greatly [see Figure 3], resulting in significant increase of MAE.

**Figure 5:**
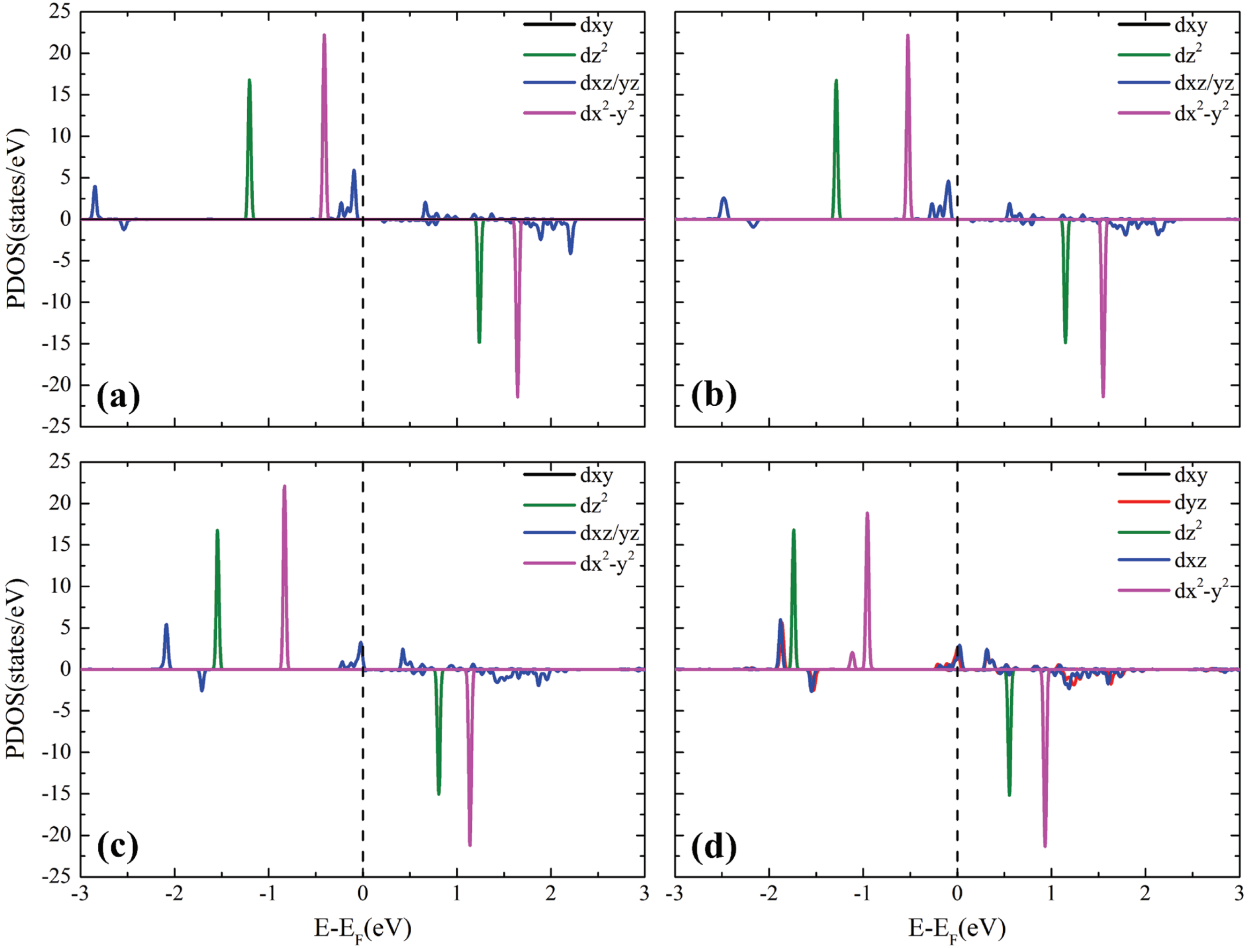
Projected density of states (PDOS) of 5d orbitals of the W atom in a) W@Pp, b) W@M‐Pp, c) W@H‐Pp, and d) W@A‐Pp.

For W@Pp based systems, the enhancement of MAE by chemical modification of functional radicals is also remarkable. **Figure** **5** shows the PDOSs of the 5d orbitals of the W atom in W@Pp based systems. By comparing the four systems, the energy level shifts of the 5d orbitals are distinct. After modification, the energy levels of the $d_{x^{2}-\;y^{2}}$ and $d_{z^{2}}$ orbitals move downward, due to the indirect repulsion from the EDGs. The magnitudes of the energy level shifts increase with increasing electron donating capacity of the functional radicals. In addition, the substitutional radicals make the *d*_*xz*/*yz*_ band become narrower and move toward the Fermi level. Consequently, the state of the *d*_*xz*/*yz*_ orbital in majority spin channel becomes partially occupied for hydroxyl and amino modified W@Pp systems, leading to significant reduction of the local spin moment on W atom (see Table 2). From the MAEs in Figure S2 of Supporting Information, we can see that the large positive MAEs are contributed from the coupling between *d*_*xz*/*yz*_ in majority spin channel and $d_{x^{2}-\;y^{2}}$ and $d_{z^{2}}$ in minority spin channel, the same as the situation in Re@Pp based systems. However, the states of the *d*_*xz*/*yz*_ orbital in W@Pp based systems are more delocalized, which reduces the contribution to MAE. Therefore, the enhancement of MAE in W@Pp based systems is less significant than that in Re@Pp based systems.

Apart from the magnetic anisotropy, another crucial factor for applications of TM@Pp is the magnetic coupling behavior. To serve as a magnetic building block in spintronics devices, ferromagnetic (FM) ground state is favorable, because antiferromagnetic (AFM) state introduces extra degree of freedom to control.^31^ Using a 2 × 2 supercell, we calculated the exchange energy (*E*_ex_) for W@Pp and Re@Pp based systems: *E*_ex_ = *E*_AFM_ − *E*_FM_, where *E*_AFM_ and *E*_FM_ denote the total energies of AFM and FM states, respectively. As shown in **Table** **3**, *W*@Pp based systems prefer AFM coupling, whereas Re@Pp based systems prefer FM coupling. Interestingly, the exchange energies of W@Pp series are closely dependent on the electron donating capacity of the functional radicals. As the electron donating capacity increases, the AFM coupling is gradually strengthened, as indicated by the increasing magnitudes of the exchange energies in Table 3. In contrast, the exchange energies of Re@Pp based systems are over 180 meV and change little with different functional radicals. Accordingly, we can estimate the Curie temperature (*T*_C_) by using the Ising model and the mean field theory (MFT).^32^, ^33^ Considering the first nearest magnetic coupling, the Hamiltonian can be written as

(3) $$H\;\;=\;\;-\;\sum_{i,j}J_{c}M_{i}\cdot M_{j}$$

**Table 3:** The exchange energy (*E*_ex_, in meV) of W@Pp and Re@Pp based systems

	TM@Pp	TM@M‐Pp	TM@H‐Pp	TM@A‐Pp
W	−34	−65	−186	−188
Re	182	183	187	186

Here, *J*_C_ is the first nearest magnetic coupling parameter, *M*_i_ is the magnetic moment at site i. Then, we can obtain *J*_C_ for the TM@Pp as

(4) Jc  =  Eex16M2

For Re@Pp based systems, the magnetic moment *M* on each site is 3 µ_B_, and *E*_ex_ is ≈185 meV, so the calculated *J*_C_ is 1.28 meV. By solving the partition function,^33^ the Curie temperature can be expressed as *T*_C_ = 5*γJ*_C_/*k*_B_, where γ is the the first nearest coordination number of the Re atoms and *k*_B_ is the Boltzmann constant. Hence, the estimated *T*_C_ is 297 K. It is known that *T*_C_ is usually overestimated by MFT. In order to get a precise *T*_C_, Monte Carlo (MC) simulations were performed to calculate the magnetic moment as a function of temperature, as shown in Figure S4 of Supporting Information. It can be seen that the newly estimated *T*_C_ is about 200 K, suggesting that the magnetism can be retained at relatively high temperature.

In addition to the stability of magnetic properties discussed above, the structural stability of TM@Pp is also crucial for applications. We performed ab initio molecular dynamics (AIMD) simulation to study the thermal dynamics of Re@Pp based systems. After AIMD run for 10 ps at 300 K, Re@H‐Pp shows slight buckling geometry with amplitude of about 1.3 Å. Nonetheless, the MAE of Re@H‐Pp in room‐temperature structure (taken as snapshot at 10 ps in Figure S3 of Supporting Information) is still 50.1 meV, decreased by only 1 meV compared to that of the 0 K structure. Similarly, the MAE of Re@A‐Pp is also robust against thermal dynamics (from 60.8 meV at 0 K to 68 meV at 300 K).

Finally, we considered the feasibility of chemical modifications in experiments. We proposed some possible reaction routes by using the widely used reagent in organic synthesis and calculated the corresponding reaction heat, as summarized in **Table** **4**. Here, the reaction heat is defined as Δ*H* = *E*(Pro) − *E*(*Rea*), where *E*(Pro) and *E*(Rea) refer to the total energies of the products and reactants, respectively. For all proposed reactions, the calculated values of Δ*H* are negative, indicating exothermic reactions. Most importantly, the reaction heat is remarkably large for the substitution of hydrogen by hydroxyl or amino, implying good feasibility for the proposed modification under proper circumstances.

**Table 4:** The proposed substitution reactions and corresponding reaction heats (Δ*H* in unit of eV)

Reaction formula	Δ*H* [eV]	
	TM = W	TM = Re
TM@Pp + 8CH_3_Cl → TM@M‐Pp + 8HCl	−1.12	−0.11
TM@Pp + 8Cl_2_ + 8H_2_O → TM@H‐Pp + 16HCl	−7.93	−6.90
TM@Pp + 8H_3_C‐NH_2_ → TM@A‐Pp + 8CH_4_	−3.89	−2.81

## 3 Conclusion

In summary, we studied the electronic and magnetic properties of 2D TM@Pp and found that both W@Pp and Re@Pp possess large perpendicular magnetic anisotropy with MAE of 24 meV, based on systematic first‐principles calculations. Furthermore, we revealed that chemical modification of functional radicals is an effective method to tune the magnetic anisotropy of W@Pp and Re@Pp. The MAE Re@Pp can be enhanced up to 51.8 and 60.8 meV once being modified with hydroxyl and amino radicals, respectively, higher than twice of the original value. This phenomenon is attributed to the charge redistribution and energy shifts of Re 5d orbitals induced by the substitutional radicals. Meanwhile, Re embedded polyporphyrin frameworks exhibit strong ferromagnetic coupling with an estimated *T*_C_ of about 200 K. Therefore, Re@H‐Pp and Re@A‐Pp are promising candidates for future applications in spintronic devices such as ultrahigh density magnetic storage.

## Conflict of Interest

The authors declare no conflict of interest.

## Supporting information

Supplementary
