# Supplementary material for: Chemically Engineering Magnetic Anisotropy of 2D Metalloporphyrin
Source: Adv Sci (Weinh). 2017 Jul 18;4(10):1700019. doi: 10.1002/advs.201700019 (PMC5644233; doi:10.1002/advs.201700019)
Supplement: Supplementary file 1 — Supplementary [file ADVS-4-na-s001.pdf]

Copyright WILEY-VCH Verlag GmbH & Co. KGaA, 69469 Weinheim, Germany, 2017.

ADVANCED SCIENCE
Open Access

Supporting Information

for *Adv. Sci.,* DOI: 10.1002/advs.201700019

Chemically Engineering Magnetic Anisotropy of 2D Metalloporphyrin

Peng Wang, Xue Jiang, Jun Hu,* and Jijun Zhao*

Supporting Information For

Chemically Engineering Magnetic Anisotropy of Two-dimensional Metalloporphyrin

Peng Wang[a], *Xue Jiang*[*a], *Jun Hu*[*b], *and Jijun Zhao*[a]

[a] Key Laboratory of Materials Modification by Laser, Ion and Electron Beams (Dalian University of Technology), Ministry of Education, Dalian 116024, China

[b] College of Physics, Optoelectronics and Energy, Soochow University, Suzhou, Jiangsu 215006, China

Corresponding Author

*Email: jiangx@dlut.edu.cn (X. Jiang); jhu@suda.edu.cn (J. Hu)

Methods

First-principles calculations were performed using an accurate frozen-core projector augmented plane-wave (PAW) method within the framework of spin-polarized density function theory (DFT), as implemented in the VASP packages.[1-3] The exchange-correlation interaction was described by the spin-polarized generalized gradient approximation (GGA) using Perdew-Burke-Ernzerhof functional.[4] To evaluate the orbital moment and MAE, the spin-orbit coupling together with the magnetic non-collinearity described by Hafner and co-workers was included into the calculations.[5-6] 2D metalloporphyrin frameworks were placed in a slab model with a vacuum (along Z direction) thickness of 15 Å sufficient to avoid the interlayer interactions. An energy cutoff of 450 eV was implemented to describe the electron wave function. Monkhorst-Pack ***k***-points meshes with density of less than $2\pi\times0.0125$ Å^{-1} along the periodic X and Y directions were used for Brillouin-zone (BZ) integration. Geometry relaxation was done without any symmetry constraint until the force on each atom was less than 0.02 eV/Å and the total energy converged to 1×10^{-5} eV. AIMD simulation was performed with a time step of 1 fs. Starting from the optimized structure, MD simulation within NVT ensemble was implemented for 10 ps to evaluate the thermal stability.

The MAE was calculated by means of self-consistent calculation with higher accuracy using the optimized structures. The MAE is defined as

$$\mathrm{MAE} = E_{tot}[\,\|\,] - E_{tot}[\perp] \qquad (1)$$

Here $E_{tot}[\,\|\,]$ and $E_{tot}[\perp]$ refer to the total energies with the magnetization direction parallel and perpendicular to the XY basal plane, respectively. For $E_{tot}[\,\|\,]$, the magnetization directions along the X(Y) axis and angular bisector of X and Y axes were both taken into account. The corresponding energies are denoted as $E_X(E_Y)$ and E_{XY}, respectively.

It is known that conventional DFT calculations usually fail to properly describe the strong correlations of the localized *d* orbitals, which may affect the description of SOC interactions and the calculated MAE values.[7] The deficiency for the self-interaction in DFT can be corrected by including a Hubbard U term.[8] We tested the MAE values with regard to the effective U values for W and Re contained systems and the results are given in Table S3 and S4. Our results show that the MAEs retain positive and large values ($\geq$ 25.7 meV) after inclusion of the U term, and sometimes they can even be enhanced by the U term.

TABLES AND FIGURES

Table S1 The adsorption energy (E_{ad}) of the TM atom on the 2D TM@Pp frameworks. Here, the adsorption energy is defined as $E_{ad} = E(TM) + E(Sub) - E(Tot)$. $E(Tot)$, $E(TM)$ and $E(Sub)$ are the energies of TM@Pp system, the individual TM atom and the pure polyporphyrin framework, respectively (all in eV). The spin moment of the individual TM atom (M_{TM}) and the total spin moment per unit cell (M_S) after adsorbing on TM@Pp frameworks (both in μ_B) are also shown in the table. The energy of an individual TM atom was calculated by placing an isolated TM atom in a $15\times15\times15$ $Å^3$ cubic cell. In the calculations, spin polarizations are all considered and the electronic self-consistency iterations stopped until the energy difference between two electronic steps was less than 1×10^{-6} eV.

TM	E(TM)	E(Sub)	E(Tot)	E_{ad}	M_{TM}	M_S
Ta	−3.459	−302.791	−318.693	12.4	3	1.9
W	−4.536	−302.791	−318.830	11.5	4	4
Re	−4.607	−302.791	−317.868	10.5	5	3
Os	−2.907	−302.791	−316.841	11.1	4	2
Ir	−1.320	−302.791	−315.608	11.5	3	1

Table S2. The adsorption energy (E_{ad}, in eV) of the TM atom on the modified 2D TM@Pp frameworks.

TM	Ta	W	Re	Os	Ir
TM@M-Pp	12.4	11.4	10.3	11.0	11.3
TM@H-Pp	12.6	11.3	10.2	11.1	11.3
TM@A-Pp	12.3	11.0	9.8	10.7	10.9

Table S3. Effective U value (U_{ef}) versus the calculated MAE (in meV) of W@Pp and the modified ones.

U_{ef}/eV	W@Pp	W@M-Pp	W@H-Pp	W@A-Pp
0	24	26	29.3	36.7
0.5	25.7	26.1	30.2	39.8
1	26.7	27.2	32.6	32.8
1.5	27.9	28.5	38.0	37.5
2	29.7	30.2	51.2	60.4

Table S4. Effective U value (U_{ef}) versus the calculated MAE (in meV) of Re@Pp and the modified ones.

U_{ef}/eV	Re@Pp	Re@M-Pp	Re@H-Pp	Re@A-Pp
0	23.9	14.3	51.8	60.8
0.5	30.5	26.0	55.5	65.7
1	33.1	30.1	52.6	58.3
1.5	31.6	28.8	45.8	49.3
2	N/A[a]	N/A[a]	N/A[a]	N/A[a]

[a] U = 2 eV leads to failure of SCF convergence.

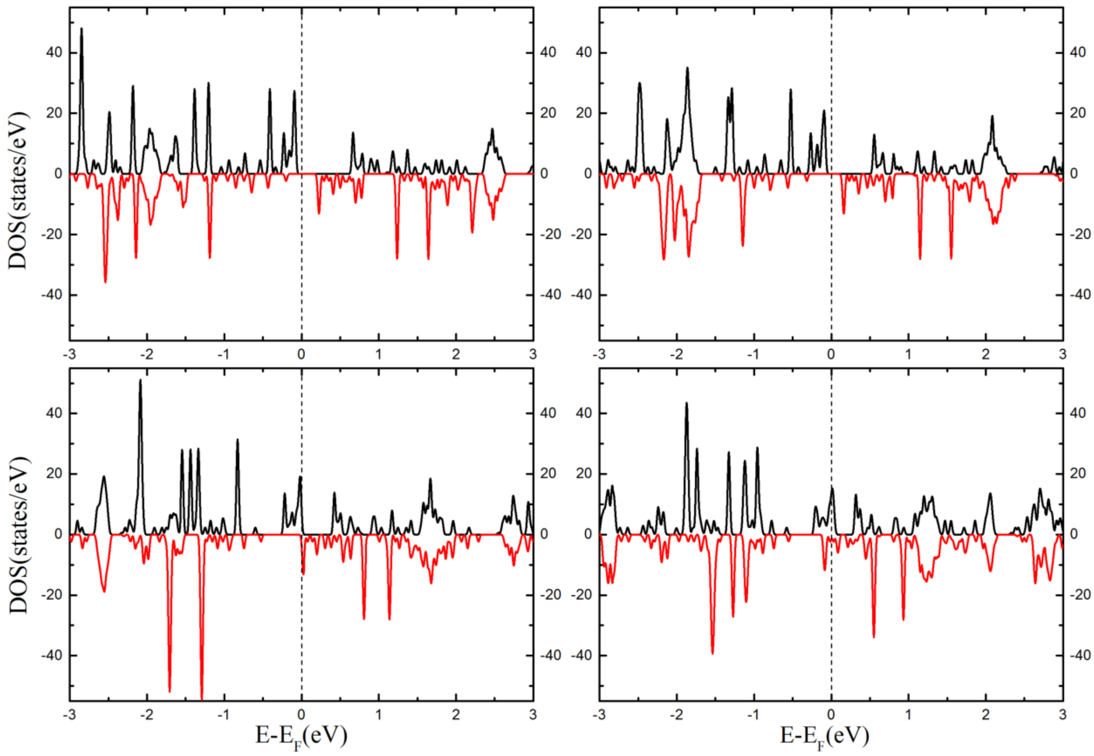

Figure S1. Total density of states (DOS) of W@Pp (a), W@M-Pp (b), W@H-Pp (c) and W@A-Pp (d). Here, black and red lines represent spin majority and minority states, respectively.

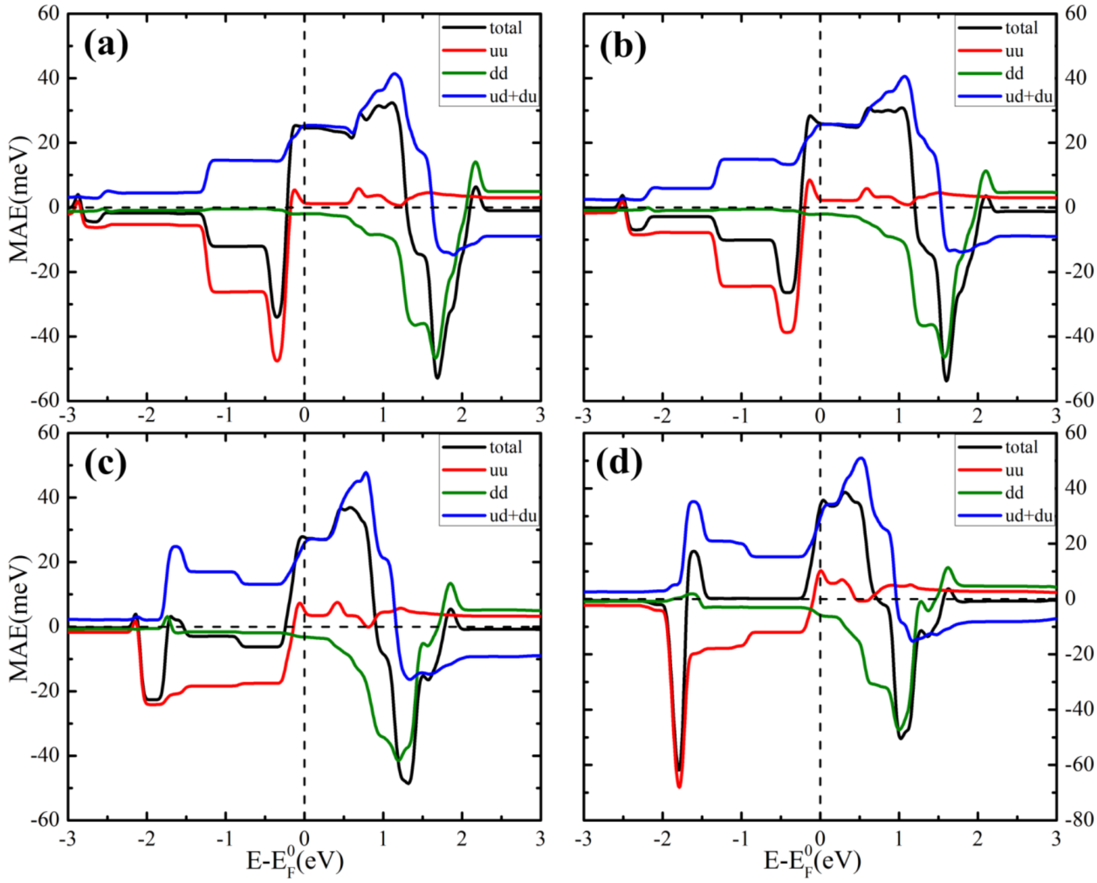

Figure S2. Fermi level dependent total and decomposed MAEs of W@Pp (a), W@M-Pp (b), W@H-Pp (c) and W@A-Pp (d). E_F^0 stands for the natural Fermi level.

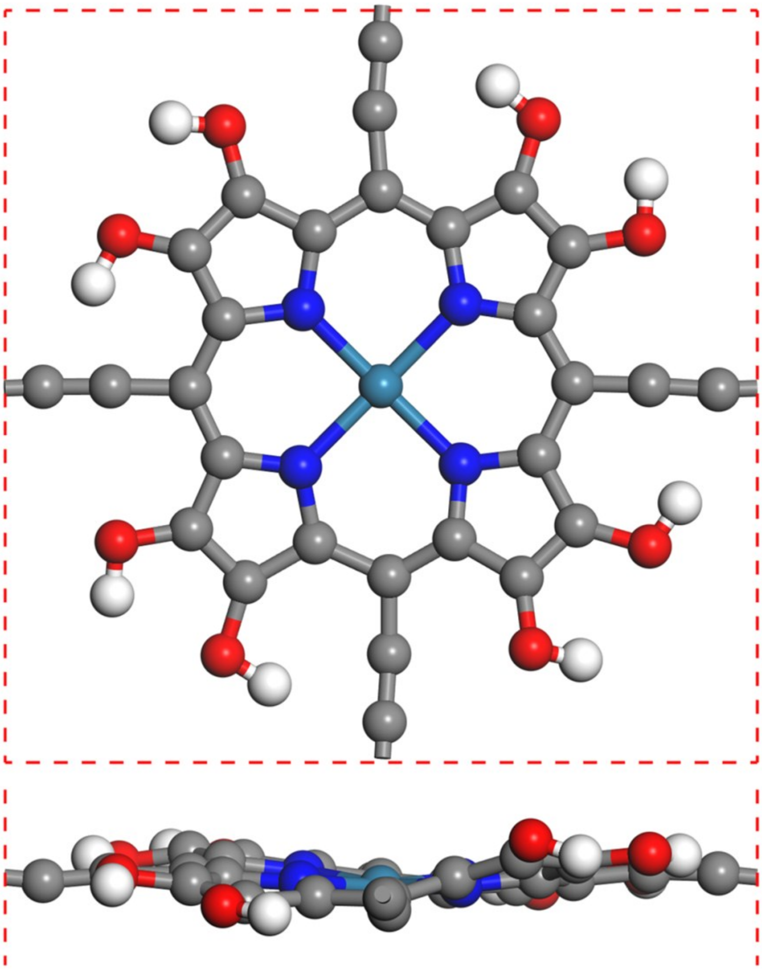

Figure S3. Top and side view of the snapshot of Re@H-Pp after 10 ps of AIMD simulation at 300K.

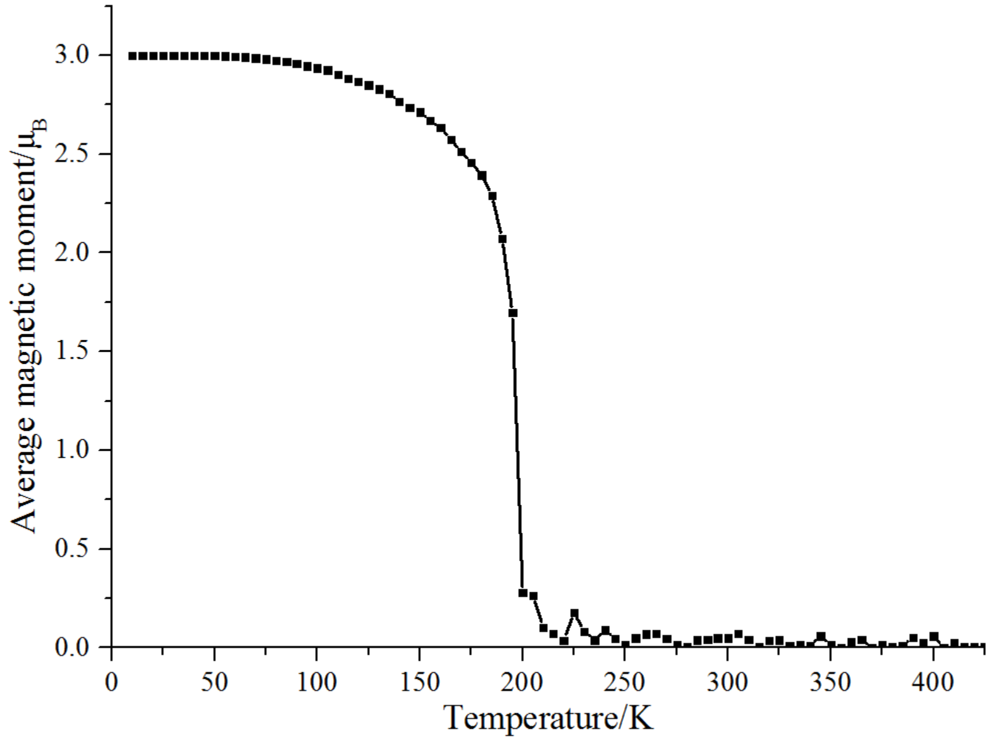

Figure S4. Variation of the magnetic moment per unit cell with respect to temperature for Re@Pp based systems obtained by MC simulations. The MC simulations last for 5×10^7 loops with a 100×100 supercell. In each loop, the spin on one randomly selected magnetic site flips randomly.

REFERENCES

(1) Kresse, G.; Hafner, J., Ab Initio Molecular Dynamics for Liquid Metals. *Phys. Rev. B* **1993**, *47*, 558.

(2) Kresse, G.; Hafner, J., Ab Initio Molecular-Dynamics Simulation of the Liquid-Metal–Amorphous-Semiconductor Transition in Germanium. *Phys. Rev. B* **1994**, *49*, 14251.

(3) Kresse, G.; Joubert, D., From Ultrasoft Pseudopotentials to the Projector Augmented-Wave Method. *Phys. Rev. B* **1999**, *59*, 1758.

(4) Perdew, J. P.; Burke, K.; Ernzerhof, M., Generalized Gradient Approximation Made Simple. *Phys. Rev. Lett.* **1996**, *77*, 3865.

(5) Hobbs, D.; Kresse, G.; Hafner, J., Fully Unconstrained Noncollinear Magnetism within the Projector Augmented-Wave Method. *Phys. Rev. B* **2000**, *62*, 11556.

(6) Marsman, M.; Hafner, J., Broken Symmetries in the Crystalline and Magnetic Structures of Γ-Iron. *Phys. Rev. B* **2002**, *66*, 224409.

(7) Błoński, P.; Hafner, J., Magnetic Anisotropy of Transition-Metal Dimers: Density Functional Calculations. *Phys. Rev. B* **2009**, *79*, 224418.

(8) Dudarev, S. L.; Botton, G. A.; Savrasov, S. Y.; Humphreys, C. J.; Sutton, A. P., Electron-Energy-Loss Spectra and the Structural Stability of Nickel Oxide: An Lsda+ U Study. *Phys. Rev. B* **1998**, *57*, 1505.
